# A peer-support lifestyle intervention for preventing type 2 diabetes in India: A cluster-randomized controlled trial of the Kerala Diabetes Prevention Program

**DOI:** 10.1371/journal.pmed.1002575

**Published:** 2018-06-06

**Authors:** Kavumpurathu R. Thankappan, Thirunavukkarasu Sathish, Robyn J. Tapp, Jonathan E. Shaw, Mojtaba Lotfaliany, Rory Wolfe, Pilvikki Absetz, Elezebeth Mathews, Zahra Aziz, Emily D. Williams, Edwin B. Fisher, Paul Z. Zimmet, Ajay Mahal, Sajitha Balachandran, Fabrizio D'Esposito, Priyanka Sajeev, Emma Thomas, Brian Oldenburg

**Affiliations:** 1 Achutha Menon Centre for Health Science Studies, Sree Chitra Tirunal Institute for Medical Sciences and Technology, Trivandrum, Kerala, India; 2 Melbourne School of Population and Global Health, University of Melbourne, Melbourne, Victoria, Australia; 3 Centre for Population Health Sciences, Lee Kong Chian School of Medicine, Nanyang Technological University, Singapore; 4 Population Health Research Institute, St George’s, University of London, London, United Kingdom; 5 Baker Heart and Diabetes Institute, Melbourne, Victoria, Australia; 6 School of Public Health and Preventive Medicine, Monash University, Melbourne, Victoria, Australia; 7 Institute of Public Health and Clinical Nutrition, University of Eastern Finland, Kuopio, Finland; 8 Collaborative Care Systems Finland, Helsinki, Finland; 9 Department of Public Health and Community Medicine, Central University of Kerala, Kasaragod, Kerala, India; 10 WHO Collaborating Centre on Implementation Research for Prevention & Control of NCDs, University of Melbourne, Melbourne, Victoria, Australia; 11 School of Health Sciences, University of Surrey, Guildford, Surrey, United Kingdom; 12 Peers for Progress and Department of Health Behavior, Gillings School of Global Public Health, University of North Carolina, North Carolina, United States of America; 13 Department of Diabetes, Central Clinical School, Monash University, Melbourne, Victoria, Australia; 14 Nossal Institute for Global Health, University of Melbourne, Melbourne, Victoria, Australia; 15 Kerala Social Security Mission, Trivandrum, Kerala, India; Centers for Disease Control and Prevention, UNITED STATES

## Abstract

**Background:**

The major efficacy trials on diabetes prevention have used resource-intensive approaches to identify high-risk individuals and deliver lifestyle interventions. Such strategies are not feasible for wider implementation in low- and middle-income countries (LMICs). We aimed to evaluate the effectiveness of a peer-support lifestyle intervention in preventing type 2 diabetes among high-risk individuals identified on the basis of a simple diabetes risk score.

**Methods and findings:**

The Kerala Diabetes Prevention Program was a cluster-randomized controlled trial conducted in 60 polling areas (clusters) of Neyyattinkara taluk (subdistrict) in Trivandrum district, Kerala state, India. Participants (age 30–60 years) were those with an Indian Diabetes Risk Score (IDRS) ≥60 and were free of diabetes on an oral glucose tolerance test (OGTT). A total of 1,007 participants (47.2% female) were enrolled (507 in the control group and 500 in the intervention group). Participants from intervention clusters participated in a 12-month community-based peer-support program comprising 15 group sessions (12 of which were led by trained lay peer leaders) and a range of community activities to support lifestyle change. Participants from control clusters received an education booklet with lifestyle change advice. The primary outcome was the incidence of diabetes at 24 months, diagnosed by an annual OGTT. Secondary outcomes were behavioral, clinical, and biochemical characteristics and health-related quality of life (HRQoL). A total of 964 (95.7%) participants were followed up at 24 months. Baseline characteristics of clusters and participants were similar between the study groups. After a median follow-up of 24 months, diabetes developed in 17.1% (79/463) of control participants and 14.9% (68/456) of intervention participants (relative risk [RR] 0.88, 95% CI 0.66–1.16, *p* = 0.36). At 24 months, compared with the control group, intervention participants had a greater reduction in IDRS score (mean difference: −1.50 points, *p* = 0.022) and alcohol use (RR 0.77, *p* = 0.018) and a greater increase in fruit and vegetable intake (≥5 servings/day) (RR 1.83, *p* = 0.008) and physical functioning score of the HRQoL scale (mean difference: 3.9 score, *p* = 0.016). The cost of delivering the peer-support intervention was US$22.5 per participant. There were no adverse events related to the intervention. We did not adjust for multiple comparisons, which may have increased the overall type I error rate.

**Conclusions:**

A low-cost community-based peer-support lifestyle intervention resulted in a nonsignificant reduction in diabetes incidence in this high-risk population at 24 months. However, there were significant improvements in some cardiovascular risk factors and physical functioning score of the HRQoL scale.

**Trial registration:**

Australia and New Zealand Clinical Trials Registry ACTRN12611000262909.

## Abstract 2

Thirunavukkarasu Sathish and colleagues report findings from a community, peer-supported trial to reduce diabetes onset in high-risk individuals in India.

## Author summary

**Why was this study done?:**

- In low- and middle-income countries (LMICs), there is an urgent need to develop low-cost strategies for identifying high-risk individuals and delivering lifestyle interventions to prevent diabetes.

**What did the researchers do and find?:**

- We conducted a cluster-randomized controlled trial in a community-setting in India to test whether a peer-support lifestyle intervention could reduce diabetes risk at 24 months.
- We identified high-risk individuals on the basis of a simple diabetes risk score.
- There was a nonsignificant reduction in diabetes risk in this high-risk population at 24 months. However, there were significant improvements in some risk factors for cardiovascular disease and a measure of quality of life.
- The intervention cost was low at US$22.5 per participant over 12 months.

**What do these findings mean?:**

- Risk scores for better identifying people at highest risk for diabetes are needed, particularly in resource-constrained settings.
- It is important to identify ways to improve program adherence and engagement, possibly by using more flexible modes of program delivery, e.g., at worksites and by text messaging.

## Introduction

Type 2 diabetes is a major public health problem worldwide [1]. Globally, an estimated 425 million people have diabetes, and the majority of those (79%) are living in low- and middle-income countries (LMICs) such as India [1]. A large proportion of people with diabetes are undiagnosed, and many present with complications at the time of diagnosis [1]. Diabetes imposes a large economic burden on individuals, their families, and national health systems [1]. Therefore, there is an urgent need to develop and implement effective and cost-effective measures to prevent diabetes.

The major efficacy trials have shown that lifestyle interventions targeting physical activity, dietary changes, and weight loss are effective [2–5] and cost-effective [6,7] in preventing type 2 diabetes among people with impaired glucose tolerance (IGT). However, while this is encouraging, the real challenge is to deliver such interventions under ‘real-world’ conditions [8]. The efficacy trials required the expensive oral glucose tolerance test (OGTT) to identify high-risk individuals and involved specialized multidisciplinary teams (e.g., physicians, nurses, dieticians, exercise physiologists) to deliver interventions. These are important factors limiting the translation of findings from the efficacy trials to real-world settings, particularly in LMICs, thereby requiring alternative strategies for identifying high-risk individuals and delivering interventions [9].

Mass screening with an OGTT to identify high-risk individuals is highly challenging in LMICs because of the cost and the limited availability of trained clinical staff and accredited laboratories [10]. Diabetes risk scores are low-cost, noninvasive, and easy-to-administer screening tools, which could reduce the number of OGTTs when used in a stepwise screening approach [11]. International guidelines and expert groups recommend using diabetes risk scores as the first screening step to identify people who may be at high risk, with blood tests undertaken to confirm high-risk status (i.e., prediabetes). These high-risk individuals can then be referred to a lifestyle intervention program [12,13]. However, even these approaches require blood testing on up to 50% of adults, posing difficulties in low-resource settings.

Lifestyle interventions evaluated in the major efficacy trials have involved resource-intensive, individualized counselling delivered on a one-to-one basis or in groups by highly trained health professionals [2–5]. In the Chinese Da Qing IGT and Diabetes Study [3], physicians delivered one individual counselling session and group counselling sessions every week for one month, followed by monthly for three months and every three months for the next 5.8 years. In the United States Diabetes Prevention Program (US DPP) [2], participants in the lifestyle intervention group received 16 individual counselling sessions from case managers within the first 24 weeks following randomization, and then, face-to-face contacts (individual or group) were made every two months for another 2.5 years. Over three years, the intervention costs were US$2,780 per participant [14]. In the Finnish Diabetes Prevention Study [5], participants received one-to-one individualized counselling sessions from nutritionists. Seven sessions were delivered in the first year and one session every three months thereafter until the end of the study at six years. Exercise physiologists guided participants to increase their physical activity through individualized resistance training sessions. In the Indian Diabetes Prevention Programme (IDPP) [4], although the intervention was less labor- and resource-intensive (US$225 per participant over three years) [6] than other efficacy trials, it was delivered on a one-to-one basis by physicians, dieticians, and social workers. These intervention strategies are not feasible for wider implementation in real-world settings in LMICs, where the burden of diabetes is substantial [1] and where the availability of highly trained health professionals for program delivery is very limited [15]. Peer support is an alternative strategy to encourage people to make and sustain healthy lifestyle changes [16]. Peer support refers to the provision of practical, social, and emotional ongoing support from nonprofessionals for complex health behaviors [17]. A recent systematic review has shown that peer support is effective in bringing behavior change in prevention and management of various health conditions, including HIV/AIDS, maternal and child health, and mental health, as well as diabetes, cardiovascular disease, and other chronic conditions [17]. Peer-support interventions are low-cost, culturally appropriate, and potentially scalable [17].

The Kerala Diabetes Prevention Program (K-DPP) was a cluster randomized controlled trial (RCT) of a peer-support lifestyle intervention implemented in a community setting in India [18]. In this paper, we aimed to examine whether the intervention could reduce diabetes incidence at 24 months among high-risk individuals identified on the basis of a diabetes risk score.

## Methods

### Ethics statement

The study was approved by the Health Ministry Screening Committee of the Government of India; ethics committees of the Sree Chitra Tirunal Institute for Medical Sciences and Technology (SCT/IEC-333/May 2011), Trivandrum, India; Monash University (CF11/0457-2011000194); and The University of Melbourne (1441736) in Australia. Written informed consent was obtained from all study participants.

### Study design

This study is reported in accordance with the Consolidated Standards of Reporting Trials guidelines for cluster RCTs (S1 Checklist) [19]. The details of the K-DPP study design have been described in detail elsewhere [18], and the protocol is available at https://link.springer.com/article/10.1186/1471-2458-13-1035. Briefly, K-DPP was a cluster RCT conducted in 60 polling areas (clusters) of Neyyattinkara taluk (subdistrict) in Trivandrum district, Kerala state. Polling areas are well-defined and identifiable locations demarcated with landmarks such as hills, rivers, roads, streets, etc. by the Election Commission of India [20]. A cluster design was chosen for the study, as the risk of contamination would otherwise be high among individuals from the same community.

### Randomization and masking

At the time of study enrolment, Neyyattinkara taluk had 603 polling areas across four legislative assembly constituencies (LACs). Although there were maps available to locate polling areas in each of the four LACs, there was no single map connecting the four LACs, which could show the contiguous polling areas across the borders of LACs. To reduce the risk of selecting contiguous polling areas, we removed 244 polling areas that were located along the borders of the four LACs and selected at random 60 of the remaining 359 polling areas. The 60 polling areas were then randomly assigned in a 1:1 ratio to the control group (received an education booklet on general lifestyle advice) or the intervention group (peer-support lifestyle intervention) by an independent statistician using a computer-generated randomization sequence. After randomization, in both the intervention and control groups, there were two contiguous polling areas. Therefore, one polling area from each pair was replaced with a nearby polling area, which were at least two kilometers apart. Participants were masked to group assignment until the completion of the baseline assessment. Field staff members administering questionnaires (no group-specific questions were included in the questionnaires) and undertaking measurements at baseline and follow-ups, along with laboratory technicians and investigators, were masked. Field staff members administering the process evaluation questionnaire to intervention participants were not masked.

### Participants

From the electoral roll of each of the 60 polling areas, 80 individuals (age 30 to 60 years) were selected randomly and were approached through home visits. Eligibility criteria included no history of diabetes or other chronic illness that might affect their participation in the trial, being literate in the local language (Malayalam), not being pregnant, and not taking medications known to affect glucose tolerance (glucocorticoids, antiretroviral drugs and antipsychotics). Those satisfying the eligibility criteria were screened using the Indian Diabetes Risk Score (IDRS), which comprises four simple parameters: age, family history of diabetes, physical activity (regular exercise or strenuous work), and waist circumference [21]. At the time of study enrolment, the IDRS was the only risk score from India that had been previously evaluated in a cohort study, with a score of ≥60 being a strong predictor of incident diabetes in Asian Indians [22]. Therefore, we chose the IDRS to screen and recruit our trial participants. Those with an IDRS score ≥60 were invited to attend a community-based clinic to undergo a 75-gm OGTT. Clinics were conducted in local neighborhoods in community buildings (e.g., schools, library halls, church halls). The OGTT was performed according to the World Health Organization (WHO) guidelines [23]. A venous blood sample was taken after an overnight fast for at least eight hours, and a second blood sample was collected two hours after oral ingestion of 75-g glucose dissolved in 250–300 ml of water. Those with fasting plasma glucose (FPG) ≥7.0 mmol/l or 2-hr plasma glucose (2-hr PG) ≥11.1 mmol/l or both were diagnosed to have diabetes based on the American Diabetes Association (ADA) criteria [12]. They were then referred to a healthcare facility and were excluded from the study. The remaining individuals were enrolled in the trial irrespective of their baseline glucose tolerance. If the participant had not fasted for the recommended time, they were asked to attend another clinic in a nearby neighborhood on a different day.

### Interventions

Detailed information on the development and cultural adaptation of the intervention program have been reported elsewhere [24,25]. Briefly, the main theory underpinning the intervention program was the Health Action Process Approach model [26] with more emphasis given to collectivistic rather than individualistic strategies during the intervention design phase. The intervention program was adapted from the Finnish Good Ageing in Lahti Region (GOAL) program [27] and the Australian Greater Green Triangle (GGT) Diabetes Prevention Project [28] through situational analysis, needs assessment, and cultural translation [24,25]. This adaptation process was guided by the Intervention Mapping Approach [29]. The program utilized the core functions of peer support identified in the US Peers for Progress Program [16] and incorporated behavior change strategies that were identified from the needs assessment study [25]. The intervention model and program were tested and further refined following piloting with two groups in 2012–2013 [18].

The 12-month peer-support program consisted of 15 group sessions: an introductory session delivered by the K-DPP team; two education sessions conducted by local experts; and 12 sessions delivered by trained lay peer leaders. An introductory session was planned to introduce the group participants to the program and its mentoring style. The needs assessment study showed that at least one peer group session per month would be optimal and feasible to deliver the intervention [25], and thus 12 peer group sessions were planned. The needs assessment study also emphasized the importance of including sessions on diabetes prevention and management by local experts, as the knowledge on these among people with prediabetes in Kerala was low [25]. Furthermore, during the pilot phase [18], peer leaders were selected from within the groups, and their level of diabetes-specific knowledge was also limited. Therefore, we decided that information would be delivered by experts through two education sessions, and the peer leaders’ role would be to help participants to translate the information into their daily lives. To minimize resources, the education sessions were delivered to participants from two to three neighborhoods within close proximity. Participants were encouraged to bring family members along to further extend the reach of the education.

#### Group sessions and community activities

All group sessions were held in the local community in a convenient neighborhood facility (e.g., schools, community halls). Sessions were conducted during weekends at a convenient time for participants. In the introductory group session (lasted for 60–90 minutes), the K-DPP team introduced the program and its benefits to the participants and their family members. The two education sessions (each lasting for half a day) were conducted by experts in the field of diabetes, nutrition, and physical activity. These sessions focused on the etiology of diabetes, risk factors, misconceptions around diabetes, and the role of lifestyle change in preventing and managing diabetes. The experts also reinforced the role and importance of peer support in behavior change and encouraged participants to attend the peer group sessions. During the introductory group session, each group selected two peer leaders (one male, one female) from among their participants with assistance from the K-DPP team. Peer leaders were identified on the basis of their willingness to lead the group, social credibility, and acceptability by the group. Peer groups consisted of 10 to 23 participants, with approximately equal numbers of males and females. The first two peer group sessions were held fortnightly, and the subsequent 10 sessions were held every month thereafter, with each session lasting 60 to 90 minutes. Each session had specific objectives and structured content to be covered, which maps onto interventions delivered in the Finnish GOAL program [27,30] and the Australian GGT project [28] (refer to S1 Table for further details).

Based on a sociobehavioral intervention model for lifestyle change [26], the peer group sessions aimed to achieve the following key lifestyle objectives:

- Increasing physical activity
- Promoting healthy eating habits
- Maintaining appropriate body weight by balancing calorie intake and physical activity
- Tobacco cessation
- Reducing alcohol consumption
- Ensuring adequate sleep

Lifestyle change strategies for increasing physical activity focused on identifying enjoyable activities for individuals and groups (e.g., walking groups, yoga sessions) and building those activities into each participant’s daily routine. Advice on healthy diet included increasing the intake of fruits and vegetables and reducing the portion size of rice and intake of fried foods and refined sugars. Participants used goal setting, action planning and self-recording of activities in their program workbook as key behavioral strategies for increasing physical activity and healthy eating. Participants received a handbook containing information on peer support, its benefits, and its role in assisting with sustainable lifestyle changes related to reducing diabetes risk. Body weight of participants was measured during the peer group sessions. Peer leaders also had regular contact with their group participants between the formal group sessions in order to reinforce the program objectives, update on the content of missed sessions, and encourage goal attainment and attendance at the next peer group session. Group participants were also encouraged by their peer leaders to participate in community activities such as establishing kitchen gardens, yoga training, and walking groups to support lifestyle change. These activities were led by peer leaders with assistance from local resource persons (LRPs) (community volunteers). While groups were encouraged to keep meeting at completion of the formal program at 12 months, there was no structured support provided for this.

#### Training, quality assurance and support for peer leaders

Peer leaders were provided two training sessions (each of two days’ duration) by the K-DPP staff members: an intervention manager (registered nurse with a PhD in public health) and an intervention assistant (medical social worker). These sessions aimed to build peer leaders’ basic knowledge about diabetes, to emphasize the role of a peer leader, and to provide skills on group facilitation, communication, goal setting, and promoting community activities. Refer to S1 Text for the content of these training sessions. Peer leaders were provided with a workbook, which outlined peer group sessions’ objectives, along with an activity guide and exercises to prepare them for conducting the sessions. Peer leaders were also given measuring cups and spoons to assist them in educating the participants about the daily recommended food quantities such as consumption of oil, sugar, salt, rice, and vegetables. Trained LRPs were asked to attend the peer group sessions as observers and to complete a detailed checklist to ensure program fidelity.

During the program delivery, peer leaders were also supported in the following ways:

- Telephone calls were made by the K-DPP team before and after each session to discuss the sessions and any challenges faced by the peer leaders.
- Practical support was provided by LRPs to help organize the logistics for local program delivery.
- Reimbursement of costs for attending training sessions was provided.

Control participants received an education booklet concerning information about diabetes and its risk factors, as well as standard advice about lifestyle change.

### Procedures

Participants were assessed at baseline, 12 months, and 24 months. During each assessment, field staff members administered standardized and validated questionnaires to collect measures of sociodemographic characteristics, lifestyle behaviors, medical history, and health-related quality of life (HRQoL). Self-reported levels of physical activity were measured using the Global Physical Activity Questionnaire [31]. Intake of fruits and vegetables were assessed using a food frequency questionnaire [32]. HRQoL was assessed using the 36-item Short-Form (SF-36) health survey [33]. The SF-36 is divided into eight scales (physical functioning, role limitation—physical, role limitation—emotional, bodily pain, general health, mental health, social functioning, and vitality) and two domains (physical component summary and mental component summary). Scores for each of the scales and domains range from 0 to 100, with higher scores indicating better quality of life. The SF-36 data were converted into a six-dimensional health state called the Short Form 6 Dimension (SF-6D), whose score ranges between 0.29 (worse health) and 1.00 (full health). Following the 12-month intervention period, a process evaluation questionnaire was administered to intervention participants by field staff members who were different from those administering main questionnaires. During each assessment, anthropometry (height, weight, fat percent, muscle mass, waist circumference, and hip circumference) and blood pressure were measured, and blood samples were taken for the OGTT, HbA1c, and lipids according to standard protocols [34]. Individuals diagnosed with diabetes on the OGTT at 12-month follow-up were referred to healthcare facilities for treatment and care. However, they were still followed-up, although an OGTT was not performed at 24 months; instead, FPG alone was measured. Blood samples were centrifuged within 30 minutes of collection and transported in boxes with dry ice to a nationally accredited laboratory. Plasma glucose was measured using the hexokinase method on a COBAS 6000 analyzer, with kits supplied from Roche Diagnostics, Switzerland. HbA1c was measured using the high-performance liquid chromatography method on a D-10 BIORAD analyzer and lipids by enzymatic methods on a COBAS 6000 analyzer, using kits supplied by Roche Diagnostics, Switzerland. Low-density lipoprotein (LDL) cholesterol was estimated using the Friedewald equation [35] for participants with triglycerides ≤4.52 mmol/l, and for the rest, values obtained from the direct method were used in the analysis.

### Outcomes

The primary outcome was the incidence of diabetes at 24 months, diagnosed by an annual OGTT, according to the ADA criteria (FPG ≥7.0 mmol/l and/or 2-hr PG ≥11.1 mmol/l) [12]. Participants who were diagnosed with diabetes by a physician and taking antidiabetic medications (‘clinical diagnosis’) subsequent to entry in the trial were also included in the primary outcome. Secondary outcomes included weight, waist circumference, waist-to-hip ratio, fat percent, muscle mass, systolic and diastolic blood pressure, FPG, 2-hr PG, HbA1c, lipid profile, IDRS score, ≥5 servings of fruit and vegetables intake per day, physical activity, tobacco use, alcohol use, and HRQoL.

### Statistical analysis

Assuming an annual incidence of diabetes of 18.3% in the control group [4], an intracluster correlation coefficient (ICC) of 0.02 for plasma glucose [36], an average of 17 participants per group, at 5% significance with 80% power, allowing a loss to follow-up of 10%, the numbers of participants and polling areas per study group required were 510 and 30 respectively, to detect a relative risk reduction (RRR) of 30% at 24 months [18]. Funding dictated that the primary outcome be measured at 24 months’ follow-up. Since ICC values for diabetes incidence were not available from published studies, ICC for plasma glucose from a previous study [36] was used. However, a positive ICC for diabetes incidence was not observed in the trial. Hence, the 32% inflation of sample size for a design effect was redundant.

Baseline characteristics of clusters and participants are summarized using mean and standard deviation (SD) or median and interquartile range (IQR) for continuous variables and frequency and percentage for categorical variables. The analyses observed intention-to-treat, i.e., participants and clusters were analyzed according to the group to which they were allocated. There were a few changes to the analysis plan specified in the study protocol [18]. For the primary outcome analysis, instead of logistic regression and Cox-proportional hazards regression, to estimate the relative risk (RR) (and 95% confidence interval [CI] and *P* value) at 24 months, we used log binomial models estimated by generalized estimating equations (GEE) with an exchangeable working correlation structure and robust standard errors to account for clustering by polling areas. This approach gave improved interpretability of the intervention effect (as increased cumulative incidence (RR) rather than increased odds) and accorded with the use of RR in the protocol’s sample size. As diabetes was only observed systematically at 12- and 24-month time points a discrete time proportional hazards model was considered appropriate in place of the Cox model but this model provides little extra information above and beyond the log-binomial model for diabetes incidence at 12 and 24 months. We also conducted post hoc subgroup analyses by baseline glucose tolerance: normal glucose tolerance (NGT), isolated impaired fasting glucose (IFG), and IGT defined by the ADA criteria [12] and the WHO criteria [23]. To examine the heterogeneity of intervention effect by subgroup, an interaction term between the intervention assignment and subgroup was included in the GEE models, and its significance was tested using the Wald test. The subgroup analyses were done because the current literature on diabetes prevention programs has been largely limited to people with IGT [37], yet the target population in the real world is much broader.

For the analysis of continuous secondary outcomes, mixed-effects linear regression models were used and included outcomes at baseline, 12 months and 24 months, and included all participants with outcome data available at one or more of these timepoints. Skewed variables were log-transformed before analysis. Study group (intervention vs. control), timepoint (follow-up vs. baseline) and a study group-by-timepoint interaction were specified as fixed effects. Random effects were specified for polling areas, to account for the clustered study design, and for participants, to account for correlation between the repeated measurements on the same individual. The *P* value of the study-group-by-timepoint interaction was used to test the difference in change between study groups. For categorical secondary outcomes, the log binomial model was used.

We assessed the sensitivity of the primary outcome analysis to missing outcome data using multilevel multiple imputation (MMI), accounting for clustering [38]. We performed 10 imputations using GEE to fit log binomial imputation models for missing outcomes and with study group and the following baseline covariates included as auxiliary variables: age, sex, education, occupation, monthly household expenditure, current tobacco use, current alcohol use, fruit and vegetable intake (in servings/day), leisure time physical activity, family history of diabetes, body mass index, waist-to-hip ratio, fat percent, muscle mass, systolic blood pressure, diastolic blood pressure, FPG, 2-hr PG, HbA1c, LDL cholesterol, and triglycerides. The log RR (and its standard error) was computed on each multiply imputed dataset, and the results were combined to obtain the multiple imputation estimate using Rubin’s rule [39]. MMI was performed using the R Jomo package [40].

The costs associated with delivering the peer-support intervention over 12 months were estimated across five major categories (training sessions for peer leaders and LRPs, group sessions, resource materials, administrative costs, and community activities). The K-DPP personnel (intervention manager and intervention assistant) were interviewed to estimate the amount of time they spent on various intervention activities. Personnel costs were calculated based on the actual salary (or remuneration) paid to the intervention manager, intervention assistant, local experts, and LRPs. Nonpersonnel costs (travel, food and logistics, rent for venues, phone calls, designing and printing charges for resource materials, and administrative costs) were estimated based on the actual expenditure. The cost figures were obtained from the finance registers. The cost estimates in Indian Rupees (INR) were converted to US dollars using an exchange rate of INR58.6 = US$1 for the year 2013 [41].

A two-sided *P* value <0.05 was considered statistically significant for all analyses. Analyses were performed using Stata version 14.2 (StataCorp LP, College Station, Texas, USA), R 3.4.3, or Microsoft Excel 2016 (Microsoft corporation, Redmond, Washington, USA).

## Results

### Participant flow and characteristics

**Fig 1:**
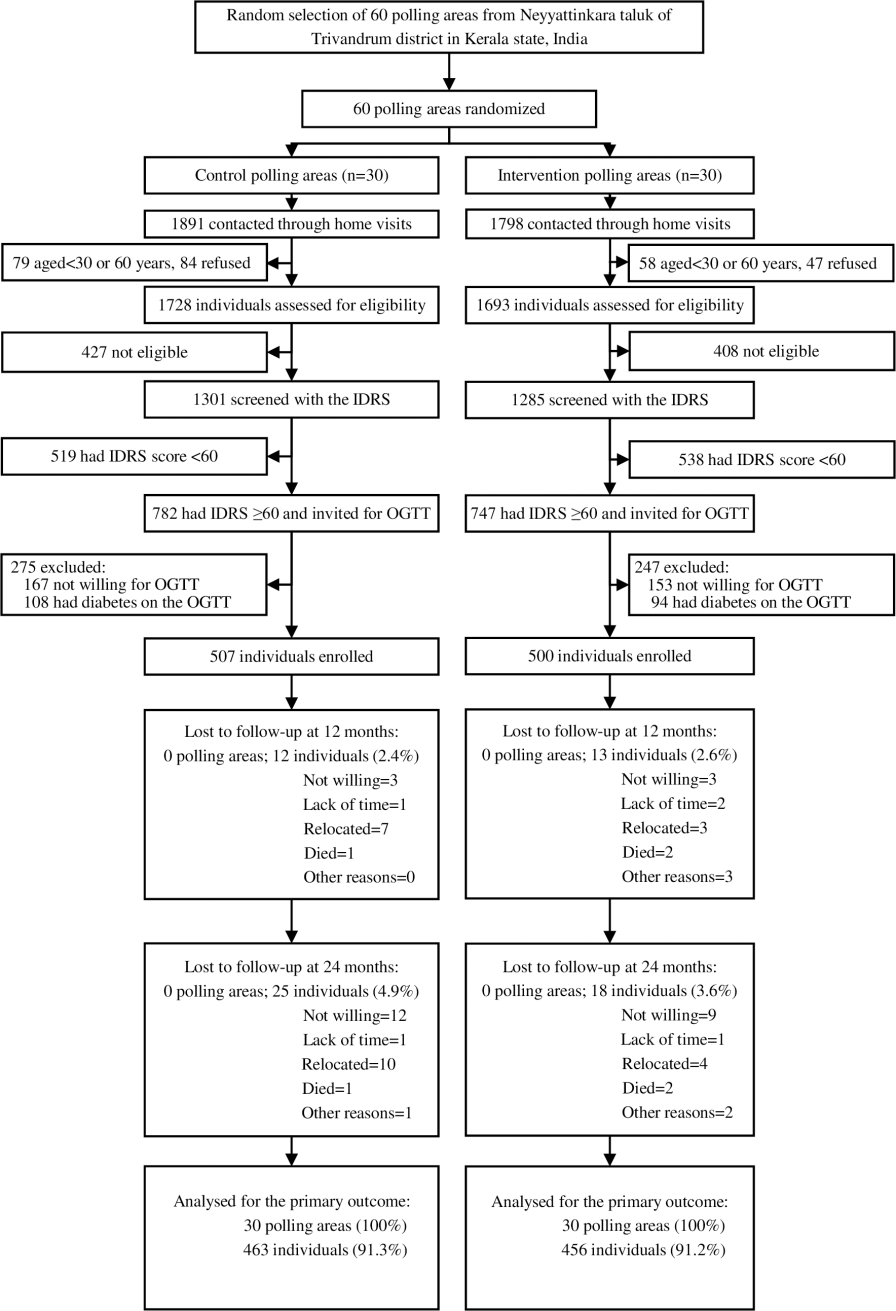
Trial profile. IDRS, Indian Diabetes Risk Score; OGTT, oral glucose tolerance test.

Participants from the 60 polling areas were recruited between January 20, 2013, and October 27, 2013. Fig 1 shows the trial profile. A total of 3,689 individuals were contacted through home visits, of whom 137 (3.7%) did not satisfy the age criteria and were therefore excluded. Of the remaining 3,552 individuals, 131 (3.7%) declined participation, and 3421 were assessed for eligibility, of whom 835 (24.4%) were not eligible. Of 2,586 eligible individuals screened with the IDRS, 1,529 (59.1%) had a score ≥60, of whom 1,209 (79.1%) attended community-based clinics and underwent an OGTT. After excluding 202 (16.7%) individuals with diabetes, 1,007 (507 in the control group and 500 in the intervention group) were enrolled in the trial. Baseline characteristics of clusters and participants were similar between the study groups (Table 1). Participants’ mean age was 46.0 years, and the majority were male (52.8%), educated up to secondary school (75.6%), and employed (72.3%). According to the ADA criteria [12], 11.5% had IGT, 57.5% had isolated IFG, and 31.0% had NGT. The corresponding figures for the WHO criteria [23] were 11.5%, 22.5%, and 66.0%, respectively. The prevalence of several cardiovascular risk factors was high at baseline, as reported previously [42]. All clusters and 95.7% (964/1007) of participants were followed-up at 24 months (95.1% in the control group; 96.4% in the intervention group).

**Table 1:** Baseline characteristics of clusters and participants by study group.

Excluded = 250: Not able to speak, read and write the local language = 40 Known T2DM = 160 Other chronic illness = 39 Pregnancy = 2 Taking medications known to influence glucose tolerance = 9	Control group	Intervention group
*Cluster level*		
No. of polling areas	30	30
Average no. of individuals/polling area	1100	1150
*Individual level*		
No. of participants	507	500
*Socio-demographic characteristics*		
Age (years), mean (SD)	45.7 (7.4)	46.2 (7.6)
Female, *n* (percent)	236 (46.6)	239 (47.8)
Education, *n* (percent) Up to primary Middle Secondary Higher secondary Vocational education College or above	117 (23.1) 143 (28.2) 123 (23.5) 42 (8.3) 31 (6.1) 51 (10.1)	136 (27.2) 129 (25.8) 114 (22.8) 43 (8.6) 28 (5.6) 50 (10.0)
Occupation, *n* (percent) Skilled/unskilled Homemaker Unemployed/retired	361 (71.2) 139 (27.4) 7 (1.4)	367 (73.4) 129 (25.8) 4 (0.8)
Monthly household expenditure (INR), median (IQR)	6000 (5000 to 10000)	7000 (5000 to 10000)
Marital status, *n* (percent) Married Separated Divorced Widowed Single	482 (95.1) 2 (0.4) 1 (0.2) 19 (3.8) 3 (0.6)	476 (95.2) 4 (0.8) 3 (0.6) 9 (1.8) 8 (1.6)
*Clinical characteristics*		
Weight (kg), mean (SD)	64.5 (12.1)	62.6 (11.6)
Waist circumference (cm), mean (SD)	88.7 (9.7)	87.9 (9.7)
Waist-to-hip ratio, mean (SD)	0.93 (0.06)	0.93 (0.07)
Fat percent (percent), mean (SD)	30.0 (8.7)	29.7 (8.2)
Muscle mass (kg), mean (SD)	42.4 (8.4)	41.5 (8.3)
Systolic blood pressure (mmHg), mean (SD)	123.4 (17.9)	123.0 (17.6)
Diastolic blood pressure (mmHg), mean (SD)	74.8 (12.1)	75.0 (11.5)
IDRS score, mean ± SD	67.5 (8.4)	66.8 (8.3)
*Bio-chemical characteristics*		
Fasting plasma glucose (mmol/l), mean (SD)	5.8 (0.5)	5.8 (0.5)
2-hr plasma glucose (mmol/l), mean (SD)	6.0 (1.5)	5.9 (1.6)
HbA1c (percent), mean (SD)	5.6 (0.5)	5.6 (0.5)
Total cholesterol (mmol/l), mean (SD)	5.7 (1.1)	5.7 (1.0)
LDL cholesterol (mmol/l), mean (SD)	3.8 (0.9)	3.8 (0.9)
Triglycerides (mmol/l), median (IQR)	1.1 (0.9 to 1.6)	1.2 (0.9 to 1.6)
*Behavioral characteristics*		
≥5 servings of fruit and vegetables/day^a^, *n* (percent)	84 (16.6)	91 (18.2)
Physically active (leisure time)^b^, *n* (percent)	102 (20.4)	107 (21.1)
Current tobacco use^c^, *n* (percent)	92 (18.2)	102 (20.4)
Current alcohol use^d^, *n* (percent)	97 (19.1)	114 (22.8)
Standard drinks of alcohol (per drinking occasion)^e^, mean (SD)	0.2 (0.4)	0.2 (0.4)
*Medical history*		
Family history of diabetes, *n* (percent)	260 (51.3)	222 (44.4)
Anti-hypertensive drugs, *n* (percent)	40 (7.9)	35 (7.0)
Lipid-lowering drugs, *n* (percent)	12 (2.4)	9 (1.8)
*Health-Related Quality of Life variables*		
Physical functioning, mean (SD)	77.8 (23.4)	75.6 (23.5)
Bodily pain, mean (SD)	70.5 (29.4)	69.9 (28.8)
Role limitation - physical, mean (SD)	83.8 (21.6)	82.2 (22.0)
Role limitation - emotional, mean (SD)	84.4 (21.9)	83.4 (23.0)
Social functioning, mean (SD)	88.1 (20.3)	87.1 (22.3)
Vitality, mean (SD)	73.2 (20.8)	74.0 (20.5)
General health, mean (SD)	64.6 (24.7)	64.9 (23.5)
Mental health, mean (SD)	76.7 (19.8)	79.4 (19.0)
Physical component summary, mean (SD)	49.9 (8.5)	49.1 (8.7)
Mental component summary, mean (SD)	53.2 (9.1)	54.0 (9.1)
SF-6D, mean (SD)	0.78 (0.15)	0.78 (0.15)

**Abbreviations:** IDRS, Indian Diabetes Risk Score; INR, Indian Rupees; IQR, interquartile range; LDL, low density lipoprotein; SD, standard deviation; SF-6D, Short Form 6 Dimension. Percentages may not add up to 100% due to rounding.

^a^ One serving of fruit equals a medium-sized fruit or two small-sized fruits or half a glass of fruit juice or a bowl of grapes. One serving of vegetables (excludes tubers) equals 80 grams.

^b^ Self-reported history of moderate or vigorous physical activities during leisure time performed in bouts of at least 10 minutes’ duration.

^c^ Smoking or use of smokeless tobacco products (chewing tobacco and snuff) in the past 30 days.

^d^ Drank an alcoholic drink in the past 30 days.

^e^ One standard drink of alcohol refers to 30 ml of spirits, 120 ml of wine, 285 ml of beer, or 285 ml of toddy (palm wine).

### Process evaluation

Of the 15 total program sessions, participants attended a median of 9 (IQR 3 to 13) sessions; 10.8% attended all 15 sessions, 62.4% attended seven or more sessions, and 89.2% attended at least one of these sessions. Twenty-nine out of 30 groups delivered all of their 12 peer group sessions, according to the intervention protocol. Nearly two-thirds (61.8%, 309/500) of intervention participants reported that they had regular contact with peer leaders outside the formal group sessions during the 12-month program, with a mean number of contacts being 11.3 (SD 8.1). More than half (57.2%, 286/500) reported participation in community activities, including yoga sessions, kitchen gardening, and walking groups. Among those who did not attend formal program sessions (*n* = 54), one-third (33.3%) still reported that they had regular contact with their peer leaders outside the group sessions, and 16.7% also reported participating in community activities. Overall, only 27 participants (5.4%) did not have any exposure to the program.

### Incidence of diabetes

After a median follow-up of 24 months, overall, diabetes developed in 147 participants (144 were diagnosed on the OGTT and 3 were clinically diagnosed): 17.1% (79/463) of participants in the control group and 14.9% (68/456) of participants in the intervention group. The RR was 0.88 (95% CI 0.66–1.16), *p* = 0.36. The RR did not change appreciably after excluding those with baseline HbA1c ≥6.5% (*n* = 52) (ADA [12] and International Expert Committee [43] cutoff value for diabetes) (RR 0.93, 95% CI 0.66–1.31, *p* = 0.66) and was similar to the results obtained using MMI (RR 0.86, 95% CI 0.61–1.13), *p* = 0.29.

The RR in the IGT subgroup (ADA and WHO criteria: 0.66, 95% CI 0.45–0.98, *p* = 0.038) was lower than that in the isolated IFG (ADA criteria: 0.95, 95% CI 0.68–1.33, *p* = 0.77; WHO criteria: 0.98, 95% CI 0.68–1.42, *p* = 0.92) or NGT (ADA criteria: 1.23, 95% CI 0.36–4.26; WHO criteria: 1.15, 95% CI 0.66–2.01, *p* = 0.63) subgroups. However, there was no evidence in favor of an interaction between baseline glucose tolerance and study group on diabetes incidence (ADA criteria: *p* = 0.24; WHO criteria: *p* = 0.11).

### Secondary outcomes

Table 2 shows the changes in clinical and biochemical characteristics by study group at 24 months. The IDRS score reduced in both study groups, but the reduction was greater in the intervention group by 1.50 points (*p* = 0.022). Table 3 shows the changes in behavioral characteristics by study group at 24 months. Compared with the control participants, intervention participants were more likely to consume ≥5 servings of fruit and vegetables per day by 83% (p = 0.008). Intervention participants were 23% less likely to consume alcohol compared with the control participants (*p* = 0.018), and the amount of alcohol consumed was lower among intervention participants (*p* = 0.030). Table 4 shows the changes in HRQoL variables by study group at 24 months. Compared with the control group, the intervention participants had a greater increase in physical functioning score of the HRQoL scale by 3.9 points (*p* = 0.016). The 12-month changes in secondary outcomes by study group are given in S2, S3 and S4 Tables.

**Table 2:** Changes in clinical and biochemical characteristics at 24 months by study group.

	Control group	Intervention group		
	Mean change (SD)	Mean change (SD)	Difference* (95% CI)	*P* value
Weight (kg)	1.24 (2.91)	1.22 (3.27)	0.01 (−0.34 to 0.36)	0.95
Waist circumference (cm)	0.63 (6.97)	−0.07 (5.95)	−0.67 (−1.56 to 0.22)	0.14
Waist-to-hip ratio	−0.011 (0.062)	−0.020 (0.078)	−0.008 (−0.018 to 0.002)	0.12
Fat percent (percent)	0.54 (3.17)	0.61 (2.77)	0.10 (−0.29 to 0.48)	0.62
Muscle mass (kg)	0.52 (3.43)	0.42 (1.69)	−0.10 (−0.39 to 0.20)	0.52
Fasting plasma glucose (mmol/l)	0.230 (0.988)	0.225 (0.811)	0.014 (−0.088 to 0.115)	0.79
Two-hour plasma glucose (mmol/l)	0.47 (2.11)	0.43 (1.97)	−0.07 (−0.34 to 0.20)	0.63
HbA1c (percent)	0.056 (0.603)	−0.003 (0.430)	−0.058 (−0.122 to 0.006)	0.08
Systolic blood pressure (mmHg)	0.28 (13.29)	−0.92 (13.44)	−1.22 (−2.80 to 0.35)	0.13
Diastolic blood pressure (mmHg)	−0.49 (9.74)	−1.59 (9.86)	−1.12 (−2.29 to 0.05)	0.06
Total cholesterol (mmol/l)	−0.11 (0.87)	−0.13 (0.80)	−0.01 (−0.11 to 0.08)	0.79
LDL cholesterol (mmol/l)	−0.09 (0.79)	−0.11 (0.73)	−0.02 (−0.11 to 0.07)	0.73
Triglycerides (mmol/l)^†^	1.11 (46.22)	1.06 (44.99)	0.96 (0.91 to 1.00)	0.07
IDRS score	−4.36 (9.61)	−5.74 (10.86)	−1.50 (−2.78 to −0.22)	0.022

**Abbreviations:** CI, confidence interval; IDRS, Indian Diabetes Risk Score; LDL, low density lipoprotein; SD, standard deviation.

* Mixed-effects linear regression was used to estimate the difference in change between study groups.

^†^ Geometric mean (coefficient of variation) is presented for within-group change and geometric mean ratio for between-group change for skewed triglycerides.

**Table 3:** Changes in behavioral characteristics at 24 months by study group.

	Control group	Intervention group	Relative risk* (95% CI)	*P* value
	(n/N [percent])	(n/N [percent])		
≥5 servings of fruit and vegetables/day^a^				
Baseline	77/507 (15.2)	68/500 (13.6)	0.90 (0.63 to 1.28)	0.56
24 months	41/482 (8.5)	67/482 (13.9)	1.83 (1.17 to 2.84)	0.008
Physically active (leisure time)^b^				
Baseline	107/507 (21.1)	102/500 (20.4)	0.94 (0.67 to 1.33)	0.73
24 months	87/482 (18.1)	101/482 (21.0)	1.20 (0.81 to 1.79)	0.36
Current tobacco use^c^				
Baseline	92/507 (18.2)	102/500 (20.4)	1.12 (0.81 to 1.55)	0.50
24 months	80/482 (16.6)	72/482 (14.9)	0.79 (0.60 to 1.05)	0.11
Current alcohol use^d^				
Baseline	97/507 (19.1)	114/500 (22.8)	1.19 (0.87 to 1.62)	0.29
24 months	88/482 (18.3)	81/482 (16.8)	0.77 (0.62 to 0.95)	0.018
	**Mean (SD)**	**Mean (SD)**	**Difference**^†^ **(95% CI)**	***P* value**
Standard drinks of alcohol (per drinking occasion)^e^				
Baseline	0.20 (0.44)	0.23 (0.43)	0.028 (−0.032 to 0.088)	0.36
24 months	0.18 (0.39)	0.17 (0.37)	−0.044 (−0.083 to −0.004)	0.030

**Abbreviations:** CI, confidence interval; SD, standard deviation.

* Generalized estimating equations was used to estimate the relative risk (and 95% CI).

^†^ Mixed-effects linear regression was used to estimate the difference in mean change between study groups.

^a^ One serving of fruit equals a medium-sized fruit or two small-sized fruits or half a glass of fruit juice or a bowl of grapes. One serving of vegetables (excludes tubers) equals 80 grams.

^b^ Self-reported history of moderate or vigorous physical activities during leisure time performed in bouts of at least 10 minutes’ duration.

^c^ Smoking or use of smokeless tobacco (chewing tobacco and snuff) in the past 30 days.

^d^ Consumed an alcoholic drink (spirits, wine, beer or toddy [palm wine]) in the past 30 days.

^e^ One standard drink of alcohol refers to 30 ml of spirits, 120 ml of wine, 285 ml of beer, or 285 ml of toddy (palm wine).

**Table 4:** Changes in health-related quality of life variables at 24 months by study group.

	Control group	Intervention group		
	Mean change (SD)	Mean change (SD)	Difference* (95% CI)	*P* value
Physical functioning	1.3 (25.6)	5.0 (25.7)	3.9 (0.7 to 7.0)	0.016
Bodily pain	4.5 (33.4)	3.1 (34.3)	−1.2 (−5.3 to 2.9)	0.55
Role limitation - physical	3.3 (25.4)	4.7 (26.9)	1.4 (−1.8 to 4.5)	0.40
Role limitation - emotional	3.1 (24.2)	5.9 (26.3)	2.5 (−0.6 to 5.6)	0.11
Social functioning	2.1 (25.6)	3.1 (27.0)	1.0 (−2.1 to 4.2)	0.52
Vitality	6.1 (22.1)	6.1 (21.5)	0.3 (−2.5 to 3.0)	0.85
General health	6.2 (26.5)	5.5 (27.3)	−0.7 (−4.1 to 2.7)	0.68
Mental health	5.4 (22.8)	4.6 (21.5)	−0.9 (−3.6 to 1.8)	0.50
Physical component summary	1.1 (9.1)	1.5 (9.0)	0.4 (−0.7 to 1.5)	0.46
Mental component summary	2.4 (10.4)	2.4 (10.0)	0.02 (−1.21 to 1.26)	0.97
SF-6D	0.04 (0.16)	0.05 (0.17)	0.01 (−0.01 to 0.03)	0.30

**Abbreviations:** CI, confidence interval; SD, standard deviation; SF-6D, Short Form 6 Dimension.

* Mixed-effects linear regression was used to estimate the difference in mean change between study groups.

### Intervention costs

Table 5 shows the costs associated with delivering the peer-support intervention over 12 months. The total intervention costs amounted to US$11,225 (US$22.5 per participant). The group sessions were the largest cost contributor (52.8% of total costs), followed by designing and printing charges for resource materials (21.7%), administrative costs (13.8%), and training of peer leaders and LRPs (11.7%). Personnel costs accounted for 26.7% of the total costs. Community activities incurred no program costs.

**Table 5:** Costs of the peer-support lifestyle intervention over 12 months.

Categories	Inputs	Cost (US dollars)	Percent of total cost
**Training sessions**		**1308**	
a. Peer leader training sessions (*n* = 2)		**691**	
	Personnel cost	135	
	Travel cost	410	
	Food and logistics	144	
	Phone calls	2	
b. LRP training sessions (*n* = 5)		**617**	
	Personnel cost	49	11.7
	Travel cost	512	
	Food and logistics	53	
	Phone calls	3	
**Group sessions**		**5928**	
a. Introductory group sessions (*n* = 30)		**1636**	
	Personnel cost	453	
	Travel cost	861	
	Rent for venues, food, and logistics	321	
	Phone calls	1	
b. DPES (*n* = 14)		**999**	
	Personnel cost	198	52.8
	Travel cost	402	
	Rent for venues, food, and logistics	397	
	Phone calls	2	
c. Peer group sessions (*n* = 348)		**3293**	
	Personnel cost	2233	
	Rent for venues, food, and logistics	1036	
	Phone calls	24	
**Resource materials**^a^ (*n* = 1560)	Designing and printing charges	**2441**	21.7
**Administrative costs**		**1548**	13.8
**Community activities**^b^		**0**	0
Total costs		**11225**	100

**Abbreviations:** DPES, Diabetes Prevention Education Sessions; LRP, local resource person. Costs in Indian Rupees (INR) were converted to US$ using an exchange rate of INR58.6 = US$1 for the year 2013. Personnel costs were calculated based on the time spent by the Intervention Manager (US$2.5/hr) and Intervention Assistant (US1.1/hr) for various intervention activities, and remuneration for local experts (US$25.6/session) and LRPs (US$4.7/session).

^a^ Includes participant handbook, peer leader handbook, peer leader workbook and health education booklet.

^b^ Includes yoga sessions, kitchen garden training, and walking groups.

### Adverse events

We recorded no adverse events related to the intervention.

## Discussion

### Summary of principal findings

To our knowledge, K-DPP is the first RCT from a LMIC to evaluate the effectiveness of a peer-support lifestyle intervention delivered mainly by lay people in a community setting. This study showed that the intervention resulted in a non-significant reduction in diabetes incidence at 24 months in a high-risk population identified on the basis of a diabetes risk score. However, there were significant improvements in some cardiovascular risk factors, including IDRS score, fruit and vegetable intake, and alcohol use, and physical functioning score of the HRQoL scale.

### Comparison with other studies

The trial was powered for a 30% RRR for diabetes incidence at 24 months and observed a 12% RRR (nonsignificant), which was lower than that reported in other effectiveness trials. A meta-analysis by Ashra and colleagues assessing the effectiveness of 13 pragmatic lifestyle interventions implemented in routine clinical practice showed that the pooled estimate of RRR was 26% [44]. In the Study on Lifestyle intervention and Impaired glucose tolerance Maastricht (SLIM) study, the RRR was 58% at three years [45]. In the Joetsu Diabetes Prevention Trial, the RRR varied from 27% (nonhospitalization with diabetes education and support) to 42% (short-term hospitalization with diabetes education and support) at three years [46]. In the Spanish Diabetes in Europe—Prevention using Lifestyle, Physical Activity and Nutritional Intervention (DE-PLAN) project, which was implemented in primary care settings, the RRR was 36% at four years [47]. In a mobile phone effectiveness study conducted in India, the RRR was 36% at two years [48]. In the Diabetes Community Lifestyle Improvement (D-CLIP) translational trial conducted in India, the RRR was 32% at three years [49]. The lower effect in our study could be attributed to the following reasons. In previous studies, most (if not all) participants had IGT, while in K-DPP, participants were identified on the basis of a risk score, and the majority had isolated IFG or NGT, albeit with a high burden of cardiovascular risk factors [42]. So far, from the limited recent literature available [49–51], there is no proven intervention to reduce diabetes incidence among those with isolated IFG. Furthermore, 24 months’ follow-up may not have been long enough to allow for an intervention effect to be observable, and thus a longer-term follow-up has been planned.

In the control group, there was a decline in fruit and vegetable intake and the reported level of physical activity at 24 months. These are consistent with findings from other recent longitudinal studies conducted in Kerala, showing that the proportion of people meeting the recommended intake of fruits and vegetables and level of physical activity is continuing to decrease over time in the absence of any intervention [52,53]. There was a greater increase in physical functioning score of the HRQoL scale in the intervention group at 24 months. Previous studies have shown that improvement in HRQoL is likely to be mediated by improved physical activity and weight loss [54].

In our study, the cost of delivering the peer-support lifestyle intervention over 12 months was US$22.5 per participant, a large percentage (52.8%) of which was accounted for by the group sessions, the main mode of formal program delivery. This is less than one-third of the intervention costs incurred in IDPP (US$75 per participant per year) [6]. The lower cost could be mainly attributed to the fact that, in IDPP, health professionals (physicians, dieticians, and social workers) were involved in delivering the intervention, while in K-DPP, the intervention was delivered mainly by lay peer leaders. In IDPP, the personnel cost was US$36 per participant, whereas in K-DPP, this cost was only US$6. However, the effect size in IDPP (28.5%) was higher than that in K-DPP (12%). It is possible that resource-intensive lifestyle interventions are more effective than low-resource interventions in reducing diabetes progression, at least in the IGT population (S5 Table and S1 Fig). If the K-DPP intervention were implemented as a real-world program, the unit costs for each individual would be much lower. This is because the one-off costs (e.g., training of peer leaders and LRPs and printing charges of resource materials) would be distributed over a much larger number of individuals, and the relative travel and administrative costs would also be lower. In K-DPP, travel and administrative costs accounted for one-third (33.3%) of the total costs. This is because the K-DPP personnel and local experts had to travel to the field, spending around three hours for every return trip. However, these costs would be relatively lower in a program setting and if the program were delivered at scale. Moreover, the very important K-DPP community activities incurred no program costs, as they were led by peer leaders with assistance from LRPs.

### Strengths and limitations

The K-DPP trial has a number of strengths. The study was conducted in the Indian state of Kerala, which has a high prevalence of diabetes (approximately 20%) and several other cardiovascular risk factors [52,55,56]. The state is in the most advanced stage of epidemiological transition compared to other Indian states [57], and it is also the harbinger of the future for the rest of India in relation to the burden of chronic diseases [55,56]. Therefore, Kerala provides an ideal setting for the implementation and evaluation of a diabetes prevention program in a community setting in India. As far as we are aware, K-DPP is the first diabetes prevention trial to deliver a peer-support lifestyle intervention program mainly by trained lay people in a low- and middle-income setting. In the D-CLIP trial from Chennai, India, although peer support was provided by community volunteers, it involved a team of health coaches and fitness trainers in the delivery of intervention. Also, metformin (500 mg twice a day) was added to those at highest risk of developing diabetes [49]. Although metformin was found to be equally effective as lifestyle intervention in previous studies [2,4] and is cheap, the current evidence base supports its use only in combination with lifestyle interventions [58]. Other strengths of our trial include a very high follow-up rate at 24 months (97.5%), use of a rigorous study design, and a much better representation of women (nearly half the participants were women) compared to previous diabetes prevention trials in India [4,48,49,59]. However, there are also some study limitations. In subgroup analyses, balance of potentially confounding characteristics between the subgroups compared is not guaranteed, and the power may have been insufficient for such analyses. Data on behavioral risk factors (tobacco use, alcohol use, physical activity, and fruit and vegetable intake) were collected using questionnaires that were not validated by objective measures and are likely to be subject to response bias. It is possible that social desirability and acquiescence biases associated with the intervention may have resulted in the small differences observed in some of the behavioral outcomes at 24 months. Furthermore, we did not adjust for multiple comparison, and given the likelihood of type 1 errors [60], changes in secondary outcomes observed should be interpreted cautiously.

### Implications for policy and future research

In efficacy trials of behavioral or social interventions, recruitment of highly selected individuals, resource-intensive interventions, and close monitoring to ensure compliance will almost always overestimate the outcomes that will actually be achievable under ‘real-world’ conditions [61]. However, given that the efficacy of lifestyle interventions to prevent diabetes among high-risk individuals, particularly among those with IGT, has been quite well established, it is now important to determine their effectiveness in real-world settings, in which the target population is likely to be much broader if program participants are not recruited on the basis of clinical testing [62]. Our study findings have some important implications for policy and future research with regards to diabetes prevention in India (and perhaps also in other LMICs). First, using a risk score rather than the OGTT to identify high-risk individuals was part of our strategy to develop a low-cost diabetes prevention program. While the IDRS with a score of ≥60 identified individuals with a high burden of cardiovascular risk factors [42], the majority had isolated IFG or NGT and not IGT. The results of our subgroup analyses suggest a trend towards greater reduction in diabetes incidence among those with IGT compared to those with isolated IFG or NGT. As mentioned previously, so far, lifestyle interventions have not been shown to be effective in reducing diabetes risk among those with isolated IFG [49–51]. Further research is required to determine the optimal cutoff for the IDRS to identify those at highest risk of developing diabetes. Alternatively, risk scores that are better at picking up people with IGT could be developed. Second, given the high burden of cardiovascular risk factors in the trial population [42] and improvements observed in some of these at 24 months, it is important to evaluate the potential longer-term benefits of the intervention on both diabetes incidence and cardiovascular risk. Finally, although the K-DPP intervention was low-cost and delivered mainly by lay people in community neighborhoods with support from local self-government bodies, it is important to do more research on how to increase program adherence and engagement, possibly by using more flexible modes of program delivery, e.g., at worksites and by text messaging. This research should also consider how continued program implementation beyond the current 12-month program for group participants can be supported by developing partnerships with other kinds of community organizations or partnerships that could deliver the intervention at scale in Kerala and elsewhere in India in the future.

### Conclusions

In this low- and middle-income setting, a low-cost peer-support lifestyle intervention resulted in a nonsignificant reduction in diabetes incidence at 24 months in a high-risk population identified on the basis of a risk score. However, there were significant improvements in some cardiovascular risk factors and physical functioning score of the HRQoL scale.

## Supporting information

S1 Checklist CONSORT 2010 checklist of information to include when reporting a CRT. CRT, cluster randomized trial.
(DOCX)

S1 Table Comparison of objectives and content of the group sessions in the Finnish GOAL program and the Australian GGT Diabetes Prevention Project with those of the peer group sessions in the K-DPP. GGT, Greater Green Triangle; GOAL, Good Ageing in Lahti Region; K-DPP, Kerala Diabetes Prevention Program.
(DOCX)

S2 Table Changes in clinical and biochemical characteristics at 12 months by study group. (DOCX)

S3 Table Changes in behavioral characteristics at 12 months by study group. (DOCX)

S4 Table Changes in health-related quality of life variables at 12 months by study group. (DOCX)

S5 Table Relative risk and intervention costs in diabetes prevention lifestyle intervention trials. (DOCX)

S1 Text Objectives and content of peer leader training sessions. (DOCX)

S1 Fig Scatter plot showing the relationship between relative risk and resource intensity of lifestyle intervention in diabetes prevention trials. (DOCX)

## Abbreviations

2-hr PG: 2-hr plasma glucose
ADA: American Diabetes Association
CI: confidence interval
D-CLIP: Diabetes Community Lifestyle Improvement Program
DE-PLAN: Diabetes in Europe—Prevention using Lifestyle, Physical Activity and Nutritional Intervention
FPG: fasting plasma glucose
GEE: generalized estimating equations
GGT: Greater Green Triangle
GOAL: Good Ageing in Lahti Region
HRQoL: health-related quality of life
ICC: intracluster correlation coefficient
IDPP: Indian Diabetes Prevention Programme
IDRS: Indian Diabetes Risk Score
IFG: impaired fasting glucose
IGT: impaired glucose tolerance
INR: Indian Rupees
IQR: interquartile range
K-DPP: Kerala Diabetes Prevention Program
LAC: legislative assembly constituency
LDL: low density lipoprotein
LMIC: low- and middle-income country
LRP: local resource person
MMI: multilevel multiple imputation
NGT: normal glucose tolerance
OGTT: oral glucose tolerance test
RCT: randomized controlled trial
RR: relative risk
RRR: relative risk reduction
SD: standard deviation
SF-36: 36-item Short-Form
SF-6D: Short Form 6 Dimension
SLIM: Study on Lifestyle intervention and Impaired glucose tolerance Maastricht
US DPP: United States Diabetes Prevention Program
WHO: World Health Organization
