# Supplementary material for: A peer-support lifestyle intervention for preventing type 2 diabetes in India: A cluster-randomized controlled trial of the Kerala Diabetes Prevention Program
Source: PLoS Med. 2018 Jun 6;15(6):e1002575. doi: 10.1371/journal.pmed.1002575 (PMC5991386; doi:10.1371/journal.pmed.1002575)
Supplement: S1 Table — GGT, Greater Green Triangle; GOAL, Good Ageing in Lahti Region; K-DPP, Kerala Diabetes Prevention Program. (DOCX) [file pmed.1002575.s002.docx]

S1 Table. Comparison of objectives and content of the group sessions in the Finnish Good Ageing in Lahti Region program (GOAL) and the Australian Greater Green Triangle (GGT) Diabetes Prevention Project with those of the peer group sessions in the Kerala Diabetes Prevention Program (K-DPP).

| **Finnish GOAL [1] and Australian GGT [2]** | **K-DPP** |
| --- | --- |
| **SESSION 1**  **Objectives:**   - Learning to know each other - Rules for the group - Discussion on current beliefs: how does life-style influence health? - Introduction by the facilitator: diabetes, risk factors & development, effects, prevention Reflective discussion and re-evaluation of beliefs   **Activities:**   - Dream—where do we want to be in 12 months’ time? How to make the dream come true: goals, planning, homework and other exercises Homework assignments: monitoring own behaviour with food diary and physical activity schedule   **SESSION 2**   - Returning of food diaries - Introduction by the facilitator: prevention really works - Evaluating own behaviour: feedback from physical activity schedule, fibre and fat tests - Discussion in small groups: comparison of own habits with the diet and physical activity goals sufficient for prevention - Role model stories with features contributing to success/failure - Discussion: analysis and re-attribution of previous successful/unsuccessful experiences - Homework assignments: preparation for goal setting, monitoring physical activity and eating habits - Discussion: barriers for group work and participation   **SESSION 3**  Feedback from the physical activity schedule  Introduction by the facilitator: health effects of physical activity  Goal planning: — Discussion: are the selected goals concrete, positive, attainable, developing? — Individual task: short-term (immediate) Where, When, How, ‘equipment’ — Feedback from homework: difficult & easy situations, what to do? Goal setting Homework assignments: feedback and re-inforcement; monitoring physical activity and eating habits Possibilities for physical activity in the local community: presentation of choices and facilities  **SESSION 4**  Food choices: feedback based on findings from food diaries Introduction by the dietician: how to eat healthy?  Goal planning: — Discussion: are the selected goals concrete, positive, attainable, developing? — Individual task: short-term (immediate) Where, When, How, ‘equipment’ — Feedback from homework: difficult & easy situations, what to do? Goal setting Exercise: how to make one’s favorite food/dishes lighter? Homework assignments: positive feedback in getting social support; monitoring physical activity and eating habits  **SESSION 5**  Discussion: evaluating and refining the goals  Discussion: routines—have they already changed? Physical activity schedule, fibre and fat tests Individual task: intermediate goals (next 6 months) Exercise: how to overcome barriers, how to use resources in maintaining the behaviour changes Discussion of ways to create peer group support system Homework assignments: monitoring physical activity and eating habits  **SESSION 6**  Discussion: evaluating the goals Discussion: routines—have they already changed? Physical activity schedule, fibre and fat tests Group discussion: analysis and re-attribution of success and failure Discussion: future goals Discussion: evaluation of the group work | **PEER GROUP SESSION 1**  **Objectives:**   - Getting to know each other - Getting an insight to one’s own lifestyle and risk and the lifestyle and risks of your family - Reflection on diabetes prevention education session - Reflection on current lifestyle enablers and barriers - Be able to measure waist circumference - Develop a group agreement   **Activities:**  **Activity 1: Introduction and icebreaker**   - Participants are instructed to meet in pairs and briefly introduce their name and three positive things about themselves. Participants then introduce each other to the whole group. - Peer leader take a note of the positive things in the peer leader manual and reviews the list with the group. Discussion on how these qualities can help in the group.   **Activity 2: Rules and objectives**   - Writing a group agreement - look at examples in workbook - Setting group objectives   **Activity 3:** **Elect two peer leaders from the group**   - Describe the roles and responsibilities of peer leader - To develop a good rapport with the members - Participate in a training day with the intervention manager and K-DPP team members - Organise the venue (with the assistance and support of the K-DPP team) for the group meetings - Organise with the K-DPP team for a guest speaker to attend sessions as required - Lead the group sessions as per the schedule and facilitate group discussions - Assist in diet and physical activity strategies and goal setting for the participant and their family/friends - Promote strategies for community activation - Record information on the duration and frequency of contact with the participants - Collect evaluation information from the participants - Maintain regular contact with the K-DPP team - Participate in peer leader meetings with the K-DPP team - Make contact with individuals who don’t attend sessions and encourage them to attend   **Activity 4: Reflection on diabetes prevention education session**  This is organized by your peer leader and co-facilitated with a K-DPP staff member. In this meeting we will discuss about diabetes and its symptoms and complications. At the end of this meeting you will:   - Learn the symptoms and complications of diabetes - Take out the participant handbook and list down all the symptoms of diabetes and its complications - Now form groups of three or four and list down four close relatives or friends suffering from diabetes - And now in groups right down the symptoms and complications you have observed in them or they have told you and cross check with that in the handbook - If more has come add to what is in the handbook. - As almost all of you had experience with diabetes patients list down some five causes which your family member or friend stated as cause of diabetes - One person from each group then reports back. Common themes identified.   **PEER GROUP SESSION 2**  **Description:**  In this meeting we will discuss about diabetes and how it is managed. At the end of this meeting you will learn some tips to manage diabetes. As we have discussed in the last meeting you all have somebody close to you suffering from diabetes and you have seen how they are managing it with medicine and with some other modifications in their lives.  List down how diabetes is managed by someone close to you whom you had a chance to closely monitor.  Diabetes management includes   - Medicines - Diet - Physical activity - Sleep   Compile all the strategies stated by the groups by peer leader.  As some of you all already know managing diabetes and preventing diabetes is almost the same. We all have heard that “Prevention is better than cure” and you all know how difficult is to manage diabetes. So by following these simple management strategies we can prevent diabetes for you and your family. Let us go through some facts. How is diabetes managed?   - In type 1 and type 2 diabetes, the aim of diabetes treatment is to get your blood glucose levels as close to the normal range as possible - For people with type 1 diabetes: insulin injections every day plus leading a healthy lifestyle - For people with type 2 diabetes: healthy eating and regular physical activity may be all that is required at first, sometimes tablets and/or insulin later on   **Closing exercise:** Share on what was the most important thing for you today in this session.  **PEER GROUP SESSION 3**  **Activity 1: Reflection**  Discussion and reflection on previous session and what you have done between sessions  **Activity 2: Self-assessment on diet**   - Participants would sit in groups and recall the diet including alcohol consumption the previous usual day using the portion pictures and serving size (included in the workbook) - Participants report their self-monitoring on diet to the rest of the group and identify something positive about their own diet - Peer leader supports and encourages the group   **Activity 3: Self-assessment on physical activity**   - Participants would sit in groups and recall the physical activity on the previous usual day using pictures in the work book - Participants report their self-monitoring on physical activity to the rest of the group and identify something positive about their activity - Peer leader supports and encourages the group   **Discussion**: How did participants relate this diet and physical activity recall exercise to their family? What insights did they gain to their as well as their family members’ lifestyle?  **Activity 4:** If you think of yourself now and ten years ago, have you noticed any difference in your weight and waist circumference? How do you know if you have gained weight? Tell in your group of three ways you monitor your body weight?  Introduce the concept of waist circumference measurement using a non-elastic inch tape.  **Exercise:** Peer leader teaches the participants how to measure waist circumference. All participants provided with a tape measure.  **Home work:** Self-monitoring of diet and physical activity in the workbook.  **Closing exercise**  **PEER GROUP SESSION 4**  **Activity 1: Reflection on self-monitoring**  Participants discuss on their thoughts and ideas following the self- assessment on diet and physical activity and recognise the areas which needs to improved or sustained. They can discuss with the group members on the possible ways of improving activity. Peer leader can give suggestions on various activities that can be done   - What already works really well for me and my family? - What am I happy about in my diet and the diet of my family? - What am I happy about with my physical activity and the physical activity of my family?   **Activity 2: What is physical activity?**   - Discussion about benefits and various intensities of physical activity - Discussion about different types of physical activities they might feel comfortable doing   **Activity 3:** Identify one specific simple thing that you would be comfortable to change with reference to diet and/or physical activity from the self-monitoring. Mark it in the workbook (Doing more and doing less with pictures)   \|  \| **Diet** \| **Physical activity** \| \| --- \| --- \| --- \| \| **More** \| Pictures of FV, pulses \| Walking, household moderate activities, night time sleeping \| \| **Less** \| Oil consumption, fried food, rice portion, alcohol, sweet snacks, sugar beverages \| Sitting time, TV watching, motorised transportation, day time sleeping \|   **Closing exercise**  **PEER GROUP SESSION 5**  **Learning Objectives:**   - Identifying foods within your family that form the basis of a healthy diet - Setting diet goals - Identifying group strategies to promote healthy diet for your family and in your community   **Activity 1: Reflection from the previous session**  Participants discuss and give feedback on their experiences with simple changes that they have made after the previous session.  **Discussion:** Think of your childhood and early adulthood days, and what was dietary pattern you had during those days? What kind of role did eating play in those days when compared to the present day? How is it similar or different to the present day? What are the good things that you would like to bring back to the present life and can still be carried on?  **Activity 2: Goal setting for diet**  **Exercise:**  Planning together with family members if they are present and/or peers with same/similar goal and using resources provided by the program to help in planning e.g., identifying from participant handbook feasible options from a list of “Top choices:”– i.e., healthy, available and affordable; “Cooking tips”; “Limit/ enjoy in moderation these food items”. Again, goal planning is where family / peer support really can start to play a significant role.  **Exercise:** Discuss on the measure that each participant uses at home to take salt, sugar and oil.  To recognise different sizes of spoons and measuring cup to measure oil, sugar and salt (pictures)   \|  \| **Diet** \| \| --- \| --- \| \| **More** \| Pictures of FV, pulses \| \| **Less** \| Oil consumption, fried food, rice portion, alcohol, sweet snacks, sugar beverages \|   **Closing discussion:** How will I incorporate the plans into my daily life?  **PEER GROUP SESSION 6**  **Learning Objectives:**   - Identifying physical activities within your family that form the basis of a healthy lifestyle - Setting physical activity goals - Assess on the adequacy of sleep and find ways to promote it. - Identifying group strategies to promote active lifestyle for your family and in your community   **Activity 1: Reflection from the self-monitoring session**  Participants discuss and give feedback on their experiences with simple changes that they have made.  **Discussion:** Think of your childhood and early adulthood days, and what was physical activity and sleep pattern you had during those days? What kind of role did physical activity play in those days when compared to the present day? How is it similar or different to the present day? What about sleep? What are the good things that you would like to bring back to the present life and can still be carried on?  **Activity 2:** Assess your sleep pattern and discuss on the ways to promote to adequate level  **Activity 3:** Goal setting for physical activity   \|  \| **Physical activity** \| \| --- \| --- \| \| **More** \| Walking, household moderate activities, night sleep \| \| **Less** \| Sitting time, TV watching, motorised transportation, day sleep \|   **Exercise:** Planning together with peers with same/similar goal (e.g., mapping together walkable areas in the neighbourhood, selecting time, place, with whom to walk).  **Closing discussion:** Peer leader reviews on the plans that the group made. Discuss on the available resources in the community (such as key people, organisations and facilities in the local settings) that can be utilised to execute the plans.  **PEER-GROUP SESSION 7**  **Learning Objectives:**   - Reviewing and evaluating progress towards goals, modifying goals as appropriate - Gaining self-efficacy through successful goal attainment   **Activity 1:** Reflection on previous session and progress since last session  **Activity 2:** Evaluation   - Refer to the goal monitoring progress sheet for diet and physical activity - Discussion in pairs/small groups   - Questions: - How did you feel you are progressing with your goals? - What has helped you? - What has hindered you? - What do you think of the effort you have to put in to achieving your goals? - Who have you worked with to achieve your goals?   **Activity 3:** Reporting to the whole group, peer leader collects and records in her manual   - Goals fully achieved - Goals partly achieved - Participant feeling related to goals (took a lot of effort --- easy)   **Activity 4:** Discussion on topics related to goal pursuit   - Helping factors - Ways to guarantee goal attainment (eg., modifying the environment, informing the family members, friends)   **Activity 5:** Discussion on future activities for group.  Suggestions could include: Cooking classes, market shopping excursion- healthy food choices, yoga classes/dance classes/other type of physical activity, guest speaker on diet, guest speaker on alcohol and smoking cessation, etc.  **PEER-GROUP SESSION 8**  **Interim evaluation of participant benefits**  Possible exercise:   - Group discussion on activities that were undertaken with respect to the program (whether or not included in the goal setting) the benefits within self and among family members in terms of happiness, feeling of well-being, energetic, weight and waist circumference assessment. Report to the whole group and the peer leader can record it in the handbook.   **PEER-GROUP SESSIONS 9-11**  The next three sessions will be on topics as determined by the groups. In these sessions the groups will be encouraged to invite members of the expert panel to facilitate sessions, to involve family and friends and to think about how to utilise resources in their own community to reach their goals.  **Learning objectives of these sessions**   - Ongoing review of goals - Planning and evaluation along with the specific topics - Deepening knowledge - Building up on the community resources - Gaining self- efficacy in making and sustaining the behaviour change**.**   **Activity 1:** Goal review   - Discussion in pairs/ small groups based on goal progress evaluation sheet   Questions:   - - How did you feel you are progressing with your goals?   - What helped you to achieve your goal?   - What hindered your achievement?   - What are your plans in sustaining and upgrading your goals?   - Who have you worked with in your community to reach your goals? - Report to the peer leader on the goals attained and upgraded (Record in the peer leader manual)   **Activity 2 (options):** Working with topics based on participants’ interests/needs:   - **Healthy diet** (portion size, identifying cooking substitutions to reduce fat, increase fruit/vegetable consumption etc.   Possible exercises:   - Content briefing from handbook, Success stories, life course transition assessment, practical and feasible strategies for healthy diet, healthy cooking sessions, healthy food exhibition cum sale, vegetable cultivation, recipe modification, role plays……,) - **Approaches to increasing physical activity** (finding enjoyable activities for individual, incorporating it into daily routines, avoiding injuries/accident)   Possible exercises:   - Content briefing from handbook, Success stories, life course transition assessment, role plays, practical and feasible strategies for being active, stretching exercise, dancing session, yoga sessions) - **Tobacco control and cessation**   Self -assessment on tobacco use and goal setting can be done as optional sessions based on the felt need.  Possible exercises:   - Content briefing from handbook, Success stories, life course transition assessment, practical and feasible strategies for control and cessation, interactive sessions by a cessation clinic social worker, video clips, role plays) - **Alcohol consumption**   Self-assessment on alcohol consumption and goal setting can be done as optional sessions based on the felt need.  Possible exercises:   - Content briefing from handbook, Success stories, life course transition assessment, practical and feasible strategies for control and cessation, interactive sessions by a de-addiction clinic social worker, video clips, role plays). - **Resources that can be utilised for sustainability and support**   Possible exercises:   - Discussion with the local leaders on modifying the environment for sustainable behaviour change, awareness campaigns by the participants, healthy food exhibition cum sale, dancing session, yoga sessions. - **Weight and waist circumference could be recorded in the handbook**   **PEER-GROUP SESSION 12**  Participants will be asked to provide a final evaluation on the program. Participants will be encouraged to bring their family members or friends that have been involved in the program thus far.  **Learning Objectives:**   - To evaluate the program - To have a plan for sustainability   - Individuals behaviour   - Ongoing peer support   **Activity 1:** Final evaluation   - Assessment of program outcomes on individual and family members’ level - Goal progress and ultimate goal - Group discussion on activities that were undertaken with respect to the program (whether or not included in the goal setting) the benefits within self and among family members in terms of happiness, feeling of well- being, energetic, weight and waist circumference assessment. Report to the whole group and the peer leader can record it in the handbook - Weight and waist circumference could be recorded in the handbook   **Activity 2:** Program impact on community level  **Activity 3:** Program evaluation-questionnaire  **Activity 4:** How to maintain healthy lifestyle and continued support in daily living |

**References**

1. Uutela A, Absetz P, Nissinen A, Valve R, Talja M, Fogelholm M. Health psychological theory in promoting population health in Paijat-Hame, Finland: first steps toward a type 2 diabetes prevention study. J Health Psychol. 2004;9: 73-84.

2. Laatikainen T, Dunbar JA, Chapman A, Kilkkinen A, Vartiainen E, Heistaro S, et al. Prevention of type 2 diabetes by lifestyle intervention in an Australian primary health care setting: Greater Green Triangle (GGT) Diabetes Prevention Project. BMC Public Health. 2007;7: 249.
