# Supplementary material for: A peer-support lifestyle intervention for preventing type 2 diabetes in India: A cluster-randomized controlled trial of the Kerala Diabetes Prevention Program
Source: PLoS Med. 2018 Jun 6;15(6):e1002575. doi: 10.1371/journal.pmed.1002575 (PMC5991386; doi:10.1371/journal.pmed.1002575)
Supplement: S2 Table — (DOCX) [file pmed.1002575.s003.docx]

S2 Table. Changes in clinical and biochemical characteristics at 12 months by study group.

|  | **Control group** | **Intervention group** |  |  |
| --- | --- | --- | --- | --- |
|  | **Mean change (SD)** | **Mean change (SD)** | **Difference^*^ (95% CI)** | **P value** |
| Weight (kg) | 0.72 (2.44) | 0.46 (2.66) | -0.25 (-0.61 to 0.10) | 0.16 |
| Waist circumference (cm) | -1.10 (7.60) | -1.71 (7.59) | -0.54 (-1.43 to 0.34) | 0.23 |
| Waist-to-hip ratio | -0.022 (0.086) | -0.025 (0.099) | -0.003 (-0.013 to 0.008) | 0.62 |
| Fat percent (%) | 0.45 (3.21) | 0.41 (2.62) | -0.04 (-0.43 to 0.35) | 0.84 |
| Muscle mass (kg) | 0.33 (2.90) | 0.13 (1.69) | -0.19 (-0.49 to 0.10) | 0.21 |
| Fasting plasma glucose (mmol/l) | 0.160 (0.578) | 0.150 (0.564) | 0.001 (-0.101 to 0.103) | 0.99 |
| Two-hour plasma glucose (mmol/l) | 0.58 (2.01) | 0.45 (1.90) | -0.10 (-0.36 to 0.16) | 0.45 |
| HbA1c (%) | -0.043 (0.544) | -0.021 (0.437) | 0.034 (-0.030 to 0.099) | 0.29 |
| Systolic blood pressure (mmHg) | -1.02 (11.47) | -1.06 (12.46) | -0.03 (-1.59 to 1.53) | 0.97 |
| Diastolic blood pressure (mmHg) | -0.82 (8.79) | -0.76 (9.51) | 0.09 (-1.07 to 1.25) | 0.89 |
| Total cholesterol (mmol/l) | -0.17 (0.74) | -0.14 (0.73) | 0.04 (-0.06 to 0.14) | 0.43 |
| LDL cholesterol (mmol/l) | -0.15 (0.69) | -0.09 (0.67) | 0.07 (-0.02 to 0.16) | 0.13 |
| Triglycerides (mmol/l)^†^ | 1.09 (43.89) | 0.98 (42.22) | 0.90 (0.86 to 0.95) | <0.001 |
| IDRS score | -5.72 (9.63) | -7.13 (10.74) | -1.37 (-2.64 to -0.09) | 0.036 |

SD, standard deviation; CI, confidence interval; IDRS, Indian Diabetes Risk Score. ^*^Mixed-effects linear regression was used to estimate the difference in change between study groups. ^†^Geometric mean (coefficient of variation) is presented for within group change and geometric mean ratio for between group change for skewed variables.
