# Supplementary material for: A peer-support lifestyle intervention for preventing type 2 diabetes in India: A cluster-randomized controlled trial of the Kerala Diabetes Prevention Program
Source: PLoS Med. 2018 Jun 6;15(6):e1002575. doi: 10.1371/journal.pmed.1002575 (PMC5991386; doi:10.1371/journal.pmed.1002575)
Supplement: S3 Table — (DOCX) [file pmed.1002575.s004.docx]

S3 Table. Changes in behavioural characteristics at 12 months by study group.

|  | **Control group** | **Intervention group** | **Relative risk^*^ (95% CI)** | **P value** |
| --- | --- | --- | --- | --- |
|  | **(n/N (%))** | **(n/N (%))** |  |  |
| ≥5 servings of fruit and vegetables/day^a^ |  |  |  |  |
| Baseline | 77/507 (15.2) | 68/500 (13.6) | 0.90 (0.63 to 1.28) | 0.56 |
| 12 months | 46/495 (9.3) | 71/487 (14.6) | 1.75 (1.09 to 2.84) | 0.022 |
| Physically active (leisure time)^b^ |  |  |  |  |
| Baseline | 107/507 (21.1) | 102/500 (20.4) | 0.94 (0.67 to 1.33) | 0.73 |
| 12 months | 109/495 (22.0) | 125/487 (25.7) | 1.21 (0.84 to 1.73) | 0.30 |
| Current tobacco use^c^ |  |  |  |  |
| Baseline | 92/507 (18.2) | 102/500 (20.4) | 1.12 (0.81 to 1.55) | 0.50 |
| 12 months | 82/495 (16.6) | 73/487 (15.0) | 0.80 (0.64 to 0.98) | 0.035 |
| Current alcohol use^d^ |  |  |  |  |
| Baseline | 97/507 (19.1) | 114/500 (22.8) | 1.19 (0.87 to 1.62) | 0.29 |
| 12 months | 85/495 (17.2) | 83/487 (17.0) | 0.83 (0.67 to 1.02) | 0.08 |
|  | **Mean (SD)** | **Mean (SD)** | **Difference**^†^ **(95% CI)** | **P value** |
| Standard drinks of alcohol (per drinking occasion)^e^ |  |  |  |  |
| Baseline | 0.20 (0.44) | 0.23 (0.43) | 0.028 (-0.032 to 0.088) | 0.36 |
| 12 months | 0.18 (0.39) | 0.17 (0.38) | -0.036 (-0.075 to 0.004) | 0.08 |

SD, standard deviation; CI, confidence interval. ^*^Generalised estimating equations was used to estimate the relative risk (and 95% CI). ^†^Mixed-effects linear regression was used to estimate the difference in mean change between study groups. ^a^One serving of fruit equals to a medium sized fruit or two small sized fruits or ½ glass of fruit juice or a bowel of grapes. One serving of vegetables (excludes tubers) equals to 80 grams. ^b^Self-reported history of moderate or vigorous physical activities during leisure time performed in bouts of at least 10 mins duration. ^c^Smoking or use of smokeless tobacco (chewing tobacco and snuff) in the past 30 days. ^d^Consumed an alcoholic drink (spirits, wine, beer or toddy [palm wine]) in the past 30 days. ^e^One standard drink of alcohol refers to 30 ml of spirits, 120 ml of wine, 285 ml of beer or 285 ml of toddy (palm wine).
