# Supplementary material for: A peer-support lifestyle intervention for preventing type 2 diabetes in India: A cluster-randomized controlled trial of the Kerala Diabetes Prevention Program
Source: PLoS Med. 2018 Jun 6;15(6):e1002575. doi: 10.1371/journal.pmed.1002575 (PMC5991386; doi:10.1371/journal.pmed.1002575)
Supplement: S4 Table — (DOCX) [file pmed.1002575.s005.docx]

|  | **Control group** | **Intervention group** |  |  |
| --- | --- | --- | --- | --- |
|  | **Mean change (SD)** | **Mean change (SD)** | **Difference^*^ (95% CI)** | **P value** |
| Physical functioning | 0.6 (24.9) | 7.2 (25.7) | 6.9 (3.9 to 10.1) | <0.001 |
| Bodily pain | 1.8 (32.2) | 7.5 (33.3) | 6.0 (1.9 to 10.0) | 0.004 |
| Role physical | 0.8 (26.1) | 5.7 (25.7) | 5.0 (1.8 to 8.2) | 0.002 |
| Role emotional | 1.0 (25.8) | 6.4 (26.2) | 5.4 (2.4 to 8.5) | 0.001 |
| Social functioning | 0.7 (25.1) | 4.4 (26.1) | 3.9 (0.8 to 7.1) | 0.015 |
| Vitality | 4.5 (22.9) | 7.5 (23.8) | 3.0 (0.3 to 5.7) | 0.032 |
| General health | 6.8 (26.0) | 6.2 (26.5) | 0.9 (-2.5 to 4.3) | 0.61 |
| Mental health | 3.6 (21.5) | 4.5 (22.6) | 0.9 (-1.8 to 3.6) | 0.50 |
| Physical component summary | 0.8 (8.3) | 2.8 (8.9) | 2.1 (1.0 to 3.2) | <0.001 |
| Mental component summary | 1.5 (9.9) | 2.4 (10.5) | 0.90 (-0.33 to 2.13) | 0.15 |
| SF-6D | 0.03 (0.16) | 0.07 (0.17) | 0.04 (0.02 to 0.06) | <0.001 |

S4 Table. Changes in health-related quality of life variables at 12 months by study group.

SD, standard deviation; CI, confidence interval; SF-6D, Short Form 6 Dimension. ^*^Mixed-effects linear regression was used to estimate the difference in mean change between study groups.
