# Supplementary material for: A peer-support lifestyle intervention for preventing type 2 diabetes in India: A cluster-randomized controlled trial of the Kerala Diabetes Prevention Program
Source: PLoS Med. 2018 Jun 6;15(6):e1002575. doi: 10.1371/journal.pmed.1002575 (PMC5991386; doi:10.1371/journal.pmed.1002575)
Supplement: S5 Table — (DOCX) [file pmed.1002575.s006.docx]

S5 Table. Relative risk and intervention costs in diabetes prevention lifestyle intervention trials.

| **Study** | **Relative risk (95% CI)** | **Intervention costs**  **(per participant per year)** |
| --- | --- | --- |
| K-DPP, 2018 | 0.88 (0.66 to 1.16) | Intl $65 |
| Da Qing [1], 1997 | 0.63 (0.50 to 0.80) | No published data |
| Swinburn et al [2], 2001 | 0.76 (0.25 to 2.34) | No published data |
| DPS [3], 2001 | 0.44 (0.29 to 0.68) | No published data |
| US DPP [4], 2002 | 0.48 (0.41 to 0.58) | Intl $1029 |
| Liao et al [5], 2002 | 0.50 (0.05 to 5.21) | No published data |
| Kosaka et al [6], 2005 | 0.33 (0.10 to 1.05) | No published data |
| IDPP [7], 2006 | 0.70 (0.53 to 0.92) | Intl $206 |
| Oldroyd et al [8], 2006 | 0.70 (0.30 to 1.66) | No published data |
| SLIM [9], 2008 | 0.46 (0.22 to 0.97) | No published data |
| Kawahara et al [10], 2008 | 0.61 (0.43 to 0.88) | No published data |
| EDIPS [11], 2009 | 0.45 (0.17 to 1.22) | No published data |
| Kang et al [12], 2010 | 0.24 (0.01 to 4.10) | No published data |
| Gagnon et al [13], 2011 | 0.20 (0.01 to 3.72) | No published data |
| Sakane et al [14], 2011 | 0.53 (0.25 to 1.13) | No published data |
| Saito et al [15], 2011 | 0.68 (0.46 to 1.01) | No published data |
| Moore et al [16], 2011 | 1.99 (0.84 to 4.69) | No published data |
| Zhou et al [17], 2011 | 0.10 (0.01 to 1.76) | No published data |
| Xu et al [18], 2013 | 0.84 (0.31 to 2.27) | No published data |
| Wong et al [19], 2013 | 0.62 (0.24 to 1.61) | No published data |
| Weber et al [20], 2016 | 0.68 (0.50 to 0.93) | No published data |
| Davies et al [21], 2016 | 0.74 (0.48 to 1.14) | Intl $135 |

CI, Confidence Interval; K-DPP, Kerala Diabetes Prevention Program; DPS, Diabetes Prevention Study; DPP, Diabetes Prevention Program; IDPP, Indian Diabetes Prevention Programme; SLIM, Study on Lifestyle‐intervention and Impaired glucose tolerance Maastricht; EDIPS, European Diabetes Prevention Study. Intl $ was calculated by dividing the local currency units for the 2005 by the purchasing power parity exchange rates of the respective countries published by the World Health Organisation [22].

**References**

1. Pan XR, Li GW, Hu YH, Wang JX, Yang WY, An ZX, et al. Effects of diet and exercise in preventing NIDDM in people with impaired glucose tolerance. The Da Qing IGT and Diabetes Study. Diabetes Care. 1997;20: 537-44.
2. Swinburn BA, Metcalf PA, Ley SJ. Long-term (5-year) effects of a reduced-fat diet intervention in individuals with glucose intolerance. Diabetes Care. 2001;24: 619-624.
3. Tuomilehto J, Lindstrom J, Eriksson JG, Valle TT, Hamalainen H, Ilanne-Parikka P, et al. Prevention of type 2 diabetes mellitus by changes in lifestyle among subjects with impaired glucose tolerance. N Engl J Med. 2001;344: 1343-50.
4. Knowler WC, Barrett-Connor E, Fowler SE, Hamman RF, Lachin JM, Walker EA, et al. Reduction in the incidence of type 2 diabetes with lifestyle intervention or metformin. N Engl J Med. 2002;346: 393-403.
5. Liao D, Asberry PJ, Shofer JB, Callahan H, Matthys C, Boyko EJ. Improvement of BMI, body composition, and body fat distribution with lifestyle modification in Japanese Americans with impaired glucose tolerance. Diabetes Care. 2002;25: 1504-1510.
6. Kosaka K, Noda M, Kuzuya T. Prevention of type 2 diabetes by lifestyle intervention: a Japanese trial in IGT males. Diabetes Res Clin Pract. 2005;67: 152-162.
7. Ramachandran A, Snehalatha C, Mary S, Mukesh B, Bhaskar AD, Vijay V, et al. The Indian Diabetes Prevention Programme shows that lifestyle modification and metformin prevent type 2 diabetes in Asian Indian subjects with impaired glucose tolerance (IDPP-1). Diabetologia. 2006;49: 289-297.
8. Oldroyd JC, Unwin NC, White M, Mathers JC, Alberti KG. Randomised controlled trial evaluating lifestyle interventions in people with impaired glucose tolerance. Diabetes Res Clin Pract. 2006; 72: 117-127.
9. Roumen C, Corpeleijn E, Feskens EJ, Mensink M, Saris WH, Blaak EE. Impact of 3-year lifestyle intervention on postprandial glucose metabolism: the SLIM study. Diabet Med. 2008;25: 597-605.
10. Kawahara T, Takahashi K, Inazu T, Arao T, Kawahara C, Tabata T, et al. Reduced progression to type 2 diabetes from impaired glucose tolerance after a 2-day in-hospital diabetes educational program: the Joetsu Diabetes Prevention Trial. Diabetes Care. 2008;31: 1949-1954.
11. Penn L, White M, Oldroyd J, Walker M, Alberti KG, Mathers JC. Prevention of type 2 diabetes in adults with impaired glucose tolerance: the European Diabetes Prevention RCT in Newcastle upon Tyne, UK. BMC Public Health. 2009;9: 342.
12. Kang JY, Cho SW, Sung SH, Park YK, Paek YM, Choi TI. Effect of a continuous diabetes lifestyle intervention program on male workers in Korea. Diabetes Res Clin Pract. 2010;90: 26-33.
13. Gagnon C, Brown C, Couture C, Kamga-Ngande CN, Hivert MF, Baillargeon JP, et al. A cost-effective moderate-intensity interdisciplinary weight-management programme for individuals with prediabetes. Diabetes Metab. 2011;37: 410-418.
14. Sakane N, Sato J, Tsushita K, Tsujii S, Kotani K, Tsuzaki K, et al. Prevention of type 2 diabetes in a primary healthcare setting: Three-year results of lifestyle intervention in Japanese subjects with impaired glucose tolerance. BMC Public Health. 2011;11: 40.
15. Saito T, Watanabe M, Nishida J, Izumi T, Omura M, Takagi T, et al. Lifestyle modification and prevention of type 2 diabetes in overweight Japanese with impaired fasting glucose levels: a randomized controlled trial. Arch Intern Med. 2011;171: 1352-60.
16. Moore SM, Hardie EA, Hackworth NJ, Critchley CR, Kyrios M, Buzwell SA. Can the onset of type 2 diabetes be delayed by a group‐based lifestyle intervention? A randomised control trial. Psychology & health. 2011;26: 485‐499.
17. Zhou J. Life style interventions study on the effects of impaired glucose regulations in Shanghai urban communities. Wei Sheng Yan Jiu. 2011;40: 331-333.
18. Xu DF, Sun JQ, Chen M, Chen YQ, Xie H, Sun WJ, et al. Effects of lifestyle intervention and meal replacement on glycaemic and body-weight control in Chinese subjects with impaired glucose regulation: a 1-year randomised controlled trial. British Journal of Nutrition. 2013;109: 487-492.
19. Wong CK, Fung CS, Siu SC, Lo YY, Wong KW, Fong DY, et al. A short message service (SMS) intervention to prevent diabetes in Chinese professional drivers with pre-diabetes: a pilot single-blinded randomized controlled trial. Diabetes Res Clin Pract. 2013;102: 158-166.
20. Weber MB, Ranjani H, Staimez LR, Anjana RM, Ali MK, Narayan KM, et al. The Stepwise Approach to Diabetes Prevention: Results From the D-CLIP Randomized Controlled Trial. Diabetes Care. 2016;39: 1760-7.
21. Davies MJ, Gray LJ, Troughton J, Gray A, Tuomilehto J, Farooqi A, et al. A community based primary prevention programme for type 2 diabetes integrating identification and lifestyle intervention for prevention: the Let's Prevent Diabetes cluster randomised controlled trial. Prev Med. 2016;84: 48-56.

# World Health Organization. CHOosing Interventions that are Cost Effective (WHO-CHOICE): Purchasing Power Parity 2005. Available from: <http://www.who.int/choice/costs/ppp/en/>.
