# Supplementary material for: A peer-support lifestyle intervention for preventing type 2 diabetes in India: A cluster-randomized controlled trial of the Kerala Diabetes Prevention Program
Source: PLoS Med. 2018 Jun 6;15(6):e1002575. doi: 10.1371/journal.pmed.1002575 (PMC5991386; doi:10.1371/journal.pmed.1002575)
Supplement: S1 Text — (DOCX) [file pmed.1002575.s007.docx]

S1 Text. Objectives and content of peer leader training sessions.

**OBJECTIVES**

**Training session 1:**

1. Demonstrate an understanding of the aims and objectives of the intervention.
2. Demonstrate an understanding of the roles and responsibilities of a peer leader.
3. Demonstrate basic motivational communication skills.
4. Be familiar with the theory underpinning the intervention program.
5. Demonstrate an ability to conduct group sessions with the support of K-DPP staff.
6. Be familiar with the contents of peer leader handbook and participant handbook and strategies that can be used to guide people through the participant handbook.
7. Be able to promote activities and connections within the wider community.
8. Be able to measure waist circumference using a measuring tape and be able to teach the participants how to measure their own waist circumference.

**Training session 2:**

1. Be able to share their experiences in conducting sessions so far.
2. Be able to recognize their own strengths in leading the group and suggest on the areas they need support from the K-DPP team.
3. Be able to share the expectation and perceived needs of the group and recognize ways to improve group participation.
4. Be able to share the small changes that are made among their group members on diet, physical activity, alcohol and tobacco use.
5. Be familiar with the conduct of further sessions (Sessions 6-12).
6. Recognise the benefit of being a peer leader.

**CONTENTS**

**1. Introduction**

Activity: Discussion in pairs: each presents oneself and names three positive qualities /strengths. Each pair then introduces the other person to the whole group.

*Trainer records the response on a white board and it can be used when working on the responsibilities of the peer leader (“How these qualities / strengths will help us in becoming good peer leaders”).*

**2. Making a good group**

*This exercise serves a double purpose of establishing trust between the peer leaders as well as modelling how to promote rapport within the small group.*

Activity: Group members are asked to remember a group that they have enjoyed being with and discuss the features that make them want to be in that group. The trainer will list the characteristics coming out of the group discussion on a board. Discuss on how to make this group and their own group to have the similar characteristics.

1. **Program description**

Discussion of the program including:

- Background
- Objectives
- Program outline
- Participants benefits
- Community benefits
- Program approach in detail (i.e. interactive, motivational and empowering)

1. **Description of the roles and responsibilities of a peer leaders, K-DPP team members and local resource persons**

- Participants discuss what they think are the qualities of a good leader
- Role of K-DPP team members and local resource persons outlined to the participants
- Discuss confidentiality
- Preparing for the meetings
- Dealing with informational needs
- Setting boundaries
- Self-care

*Assimilate their views and reach a shared understanding of the qualities and responsibilities of a peer leader*

1. **Effective communication and motivational skills**

- Theory and practice on communication skills including:
  - Rapport building
  - Active listening
  - Acknowledging others views
  - Body language
  - Knowing your audience
  - Making your point succinctly
  - Roadblocks to communication
  - Managing difficult behaviours
  - Negotiating self-defeating health beliefs
- Theory and practice on motivational coaching

1. **Theory of conducting sessions**

- Program outline
- Review and discuss handbook content (peer leader and participant)
- Practise measuring waist circumference and teaching others how to measure
- Goal setting and developing an action plan
- Logistics / practical arrangements for small group sessions

1. **Practice in conducting sessions:** Mock activities from sessions 2 to 12
2. **Peer leader support and working with the research team**

Discussion about ongoing peer leader support and what the participants feel is the best way in which to provide this to them.

- Receiving updates about the research project
- Possible items in the agenda
  - Positive things about the meetings
  - Perceived benefits of being a peer leader and being a member of the group
  - Methods that peer leaders have come up with
  - What needs improvement: Finding solutions from each other
  - Community activities
  - Means taken to promote participation
  - Experiences of peer leader to peer leader interaction and support
- Possible modes of peer leader support

*Group level*:

- Amongst peer leaders (as the group decides)
- With K-DPP team

*Individual level:*

- Between two peer leaders (before and after each session)
- Between peer leader and K-DPP (telephonic contact before and after each group session)

1. **Reflection on the training program and the intervention program**

- Discussion and Q&A
