# Supplementary material for: A peer-support lifestyle intervention for preventing type 2 diabetes in India: A cluster-randomized controlled trial of the Kerala Diabetes Prevention Program
Source: PLoS Med. 2018 Jun 6;15(6):e1002575. doi: 10.1371/journal.pmed.1002575 (PMC5991386; doi:10.1371/journal.pmed.1002575)
Supplement: S1 Fig — (DOCX) [file pmed.1002575.s008.docx]

S1 Fig. Scatter plot showing the relationship between relative risk and resource-intensity of lifestyle intervention in diabetes prevention trials.

K-DPP

Davies et al 2016

US DPP

K-DPP, Kerala Diabetes Prevention Program; IDPP, Indian Diabetes Prevention Programme; US DPP, US Diabetes Prevention Program.
